# Lymphatic ERG Governs Junctional Plasticity and Fluid Clearance via an EDNRB Signaling Axis in Pulmonary Fibrosis

**DOI:** 10.64898/2026.09.17.752506

**Published:** 2026-09-23

**Authors:** Arun Narota, Tapiwa Muvavarirwa, Xintao Qiu, Adri Chakraborty, Ahmed A. Raslan, Giovanni Ligresti, Maria Trojanowska

## Abstract

Pulmonary lymphatic vessels are essential for interstitial fluid balance and macromolecular clearance, yet the endothelial mechanisms regulating lymphatic vessel function during chronic fibroproliferative lung injury remain undefined. Here, we demonstrate that the lineage-defining transcription factor ERG in lymphatic endothelial cells (LECs) governs junctional plasticity and fluid drainage in pulmonary fibrosis. In human idiopathic pulmonary fibrosis (IPF) lungs, lymphatic ERG expression is markedly downregulated. Lineage-specific inducible deletion of *Erg* in murine LECs (Erg-CKO) unexpectedly conferred robust protection against bleomycin-induced pulmonary fibrosis, significantly dampening acute inflammation, reducing edema, and preserving pulmonary compliance. Mechanistically, loss of ERG enhanced lymphatic drainage capacity in vivo. Transcriptomic profiling of isolated LECs revealed that ERG deficiency activates actomyosin contractile pathways and selectively upregulates Endothelin Receptor Type B ( *Ednrb* ), driving junctional remodeling from continuous zippers into discontinuous button-like configurations that facilitate interstitial fluid entry. Pharmacological blockade of EDNRB with BQ-788 abolished junctional reorganization, eliminated the enhanced lymphatic drainage, and completely reversed the anti-fibrotic protection in Erg-CKO mice. Together, our findings identify an endothelial ERG-EDNRB regulatory axis that controls lymphatic junctional architecture and demonstrate that promoting lymphatic clearance via endothelial junctional remodeling represents a viable therapeutic strategy for fibrotic vascular remodeling.

## Full Text Availability

The license terms selected by the author(s) for this preprint version do not permit archiving in PMC. The full text is available from the preprint server.

